# Complete Genome Sequences of the Plant Pathogens *Ralstonia solanacearum* Type Strain K60 and *R. solanacearum* Race 3 Biovar 2 Strain UW551

**DOI:** 10.1128/genomeA.01088-17

**Published:** 2017-10-05

**Authors:** Madeline M. Hayes, April M. MacIntyre, Caitilyn Allen

**Affiliations:** Department of Plant Pathology, University of Wisconsin–Madison, Madison, Wisconsin, USA

## Abstract

*Ralstonia solanacearum* is a globally distributed plant pathogen that causes bacterial wilt diseases of many crop hosts, threatening both sustenance farming and industrial agriculture. Here, we present closed genome sequences for the *R. solanacearum* type strain, K60, and the cool-tolerant potato brown rot strain *R. solanacearum* UW551, a highly regulated U.S. select agent pathogen.

## GENOME ANNOUNCEMENT

The proteobacterium *Ralstonia solanacearum* causes bacterial wilt of >200 plant species, resulting in large economic losses of diverse crops worldwide (1, 2). Because it contains many heterogeneous strains, *R. solanacearum* is considered a species complex (3). Phylogenetic analysis separates the *R. solanacearum* species complex (RSSC) into four phylotypes, which are further subdivided into several dozen sequevars. Phylotypes correspond to the strain's geographic origin Asia, phylotype I; the Americas, phylotype II; Africa, phylotype III; and Indonesia-Japan, phylotype IV) (3).

The *R. solanacearum* type strain is K60, which belongs to phylotype II, sequevar 7. K60, also known as UW25, was isolated in 1953 from a Marglobe tomato in Raleigh, North Carolina, USA (4), and has been used extensively for bacterial wilt research (5, 6). Similar sequevar 7 strains currently cause bacterial wilt of tomato, tobacco, pepper, and ornamentals in the southeastern United States (6–9). Previous sequencing of K60 generated a 547-contig draft (10). This identified over 300 K60-specific genes and contributed to phylogenetic analysis of the RSSC and its virulence factors (11). However, the low quality of this draft sequence prevented genome assembly and left significant fine-scale ambiguities in repetitive regions.

An especially destructive subgroup of the RSSC causes potato brown rot at cool temperatures (18 to 24°C) (12). Historically and for regulatory purposes known as *R. solanacearum* race 3, biovar 2 (R3bv2), strains in this subgroup mostly fall into phylotype II, sequevar 1 (3). R3bv2, which originated in the Andes like the potato, has been introduced into North America and Europe and is now endemic in some European waterways (13–15). Because it threatens potato production, R3bv2 is a highly regulated quarantine pathogen and is listed as a U.S. select agent (16, 17). The 2006 draft genome of R3bv2 strain UW551, which was isolated from a geranium plant grown in Kenya, contained 582 contigs (18). UW551 has been a model strain for exploring the survival, transmission, and cold tolerance of R3bv2 (19–22), but studies have been hindered by the lack of a closed UW551 genome. Draft genomes are available for several other R3bv2 strains, but none are closed (23–25).

Here, we present complete genome sequences of strains K60 and UW551. DNA for sequencing was extracted from overnight broth cultures using Epicentre MasterPure genomic DNA kits. PacBio sequencing to >100× coverage was performed at the Great Lakes Genomics Center (Milwaukee, WI, USA). Reads were assembled using Canu version 1.3 (26) and annotated using the NCBI Prokaryotic Genome Annotation Pipeline (27).

Both genomes contain two replicons, as is typical for *R. solanacearum*. The K60 genome encodes a predicted 4,799 genes, has a G+C content of 66.4%, and totals 5,770,663 bp in two contigs (3,383,865-bp chromosome and 1,931,798-bp megaplasmid). The UW551 genome encodes a predicted 4,551 genes, has a G+C content of 66.6%, and totals 5,478,976 bp in two contigs (3,471,163-bp chromosome and 2,007,813-bp megaplasmid).

As expected for strains within one phylotype, genes present in both K60 and UW551 have 96.07% average nucleotide identity (ANI) (28). However, they have >92% ANI to closed genomes of representative RSSC strains in other phylotypes (phylotype I GMI1000, phylotype III CMR15, and phylotype IV PSI07).

### Accession number(s).

These genome sequences have been deposited in GenBank under accession no. NCTK00000000 (K60) and NCTI00000000 (UW551).
